# Dilatation Eustachian tuboplasty with a Eustachian tube video endoscope and supporting balloon

**DOI:** 10.1017/S0022215123001202

**Published:** 2024-03

**Authors:** Huasong Zhang, Qing Zhang, Kunwu He, Minqi Chen, Yucheng Chen, Dongliang Su, Haobin Tang, Weifen Lin, Shuhua Chen

**Affiliations:** 1 Department of Otolaryngology, Second People's Hospital of Foshan (Affiliated Foshan Hospital of Southern Medical University), Foshan, China; 2 Department of Otolaryngology, Guangdong Provincial Key Laboratory of Major Obstetric Diseases, Guangdong Provincial Clinical Research Center for Obstetrics and Gynecology, Third Affiliated Hospital of Guangzhou Medical University, Guangzhou, China; 3 Department of Otolaryngology, Longgang ENT Hospital and Shenzhen Key Laboratory of ENT, Institute of ENT, Shenzhen, China; 4 School of Medicine, University of Central Lancashire, Preston, Lancashire, UK

**Keywords:** Dilation, Eustachian tube, visual endoscope, supporting balloon, feasibility, safety

## Abstract

**Objective:**

To evaluate the feasibility and safety of employing a Eustachian tube video endoscope with a supporting balloon as a viable treatment and examination option for patients with Eustachian tube dysfunction.

**Methods:**

A study involving nine fresh human cadaver heads was conducted to investigate the potential of balloon dilatation Eustachian tuboplasty using a Eustachian tube video endoscope and a supporting balloon catheter. The Eustachian tube cavity was examined with the Eustachian tube video endoscope during the procedure, which involved the dilatation of the cartilaginous portion of the Eustachian tube with the supporting balloon catheter.

**Results:**

The utilisation of the Eustachian tube video endoscope in conjunction with the supporting balloon catheter demonstrated technical ease during the procedure, with no observed damage to essential structures, particularly the Eustachian tube cavity.

**Conclusion:**

This newly introduced method of dilatation and examination of the Eustachian tube cavity using a Eustachian tube video endoscope and the supporting balloon is a feasible, safe procedure.

## Introduction

The Eustachian tube serves as the sole channel for middle-ear ventilation and drainage,^1–3^ playing a crucial role in regulating middle-ear pressure, clearing the middle-ear space and preventing diseases.^4,5^ Hence, the Eustachian tube plays an important role in the middle ear.

Eustachian tube dysfunction, which includes obstructive Eustachian tube dysfunction, baro-challenge-induced Eustachian tube dysfunction and patulous Eustachian tube dysfunction,^4^ can persist into adulthood. Such dysfunction has various causes, such as anatomical derangements, chronic sinusitis, allergic rhinitis, adenoid hypertrophy and gastroesophageal reflux.^1^ The incidence rate in adults is approximately 5 per cent,^6–8^ and 70 per cent of children experience at least one acute episode of Eustachian tube dysfunction before the age of 10 years,^9,10^ indicating the significant impact of Eustachian tube dysfunction on health.

Various medical and surgical treatments have been explored for obstructive Eustachian tube dysfunction, including balloon dilatation of the Eustachian tube (balloon dilatation Eustachian tuboplasty), laser Eustachian tuboplasty^11^ and topical application of medications.^12^ Among these treatments, balloon dilatation Eustachian tuboplasty has shown promising effects.^13,14^ Indications for balloon dilatation Eustachian tuboplasty may include: symptoms such as aural fullness lasting for more than three months; a type B/C tympanogram; a Eustachian Tube Dysfunction Questionnaire-7 (‘ETDQ-7’) score of more than 2; and the failure of medical treatment.^15^ Previous studies have demonstrated that balloon dilatation Eustachian tuboplasty can improve Eustachian tube function, based on subjective and objective measurements in patients with Eustachian tube dysfunction.^16^ Additionally, balloon dilatation Eustachian tuboplasty appears to have no significant impact on taste function, but may improve olfactory function.^17^ However, the lack of high-level evidence to support its use has led to some ENT consultants in the UK not practising balloon dilatation Eustachian tuboplasty.^18^ We believe that the existing balloon dilatation Eustachian tuboplasty techniques lack accurate operation markers and may not be performed at the optimal position of the balloon.

In this study, we designed a Eustachian tube video endoscope and a supporting balloon, and examined the feasibility of video balloon dilatation Eustachian tuboplasty in cadavers. This study aimed to assess the safety and to optimise the clinical application of this novel technique.

## Materials and methods

### Cadaver study

Ethical approval for this study was obtained from the Ethical Committee of the Second People's Hospital of Foshan (approval number: KJ2022007). In order to assess the feasibility of using the Eustachian tube video endoscope and a supporting balloon for accurate positioning in the cartilaginous portion of the Eustachian tube, we developed a procedure for balloon dilatation Eustachian tuboplasty performed under video guidance using the Eustachian tube video endoscope.

A total of nine Eustachian tubes from nine cadavers, aged 30–70 years, were included in the study, with an approximately equal distribution of left (*n* = 4) and right (*n* = 5) Eustachian tubes. All nine cadaver Eustachian tubes had maintained their shape and integrity, and were filled with colophony glue. Prior to the procedure, the cadaver specimens were fully thawed to avoid damage to the Eustachian tube mucosa and to prevent adverse effects during the operation. The cadaver specimens were selected for this study after being filled with colophony glue, to aid in assessing any damage during the procedure.

**Figure 1.**
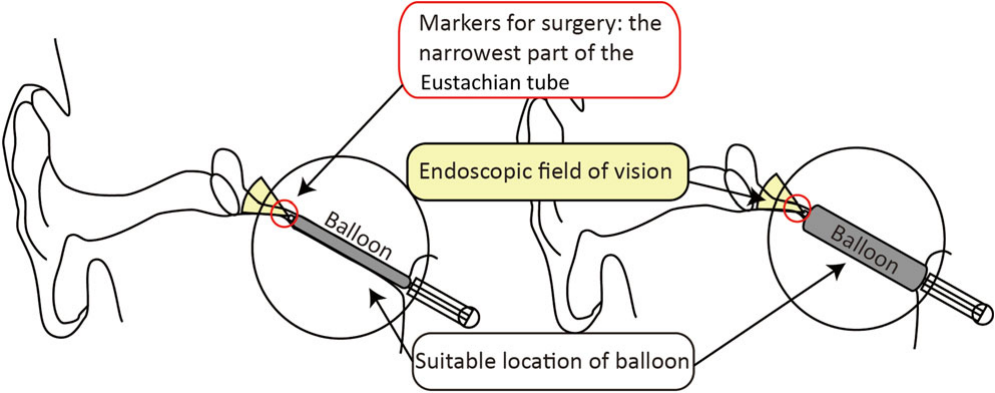
Operating principle of Eustachian tube video endoscopy. The red rings represent the surgical markers of the Eustachian tube video endoscope and supporting balloon technique. The yellow areas indicate the field of vision of the Eustachian tube video endoscope. The grey areas represent the supporting balloon of the Eustachian tube video endoscope. The black rings indicate the main operative area of the Eustachian tube video endoscope.

The Eustachian tube video endoscope used in this study was a 1.2 mm diameter, soft segment endoscope (Shaanxi FeiMiao Medical Equipment, Xi`an, China). A supporting balloon, sheathed outside the Eustachian tube video endoscope, had a diameter of 3.5 mm and a length of 16 mm, which was advanced through the working channel of the Eustachian tube video endoscope to ensure accurate positioning of the balloon in the video (Figure 1). Once the narrowest position of the Eustachian tube was visualised, the supporting balloon was inflated to a pressure of 10 bars (7.501 mmHg) for 2 minutes using a pressure applicator (Shaanxi FeiMiao Medical Equipment).

Exclusion criteria for cadavers were as follows: (1) severely narrow nasal cavity, abnormal development of the nasal cavity or the Eustachian tube, hyperplasia, or cancer, or severely deformed nasal cavity or Eustachian tube associated with injury before death; and (2) unknown source of the cadaver head, or the presence of active infectious disease.

### Efficacy evaluation

#### Procedure is highly effective

The advancement of the supporting balloon is smooth, with simple handling required. The internal tissue of the human body where the lens reaches can be clearly observed during the endoscope push process, allowing for adjustment of the push direction based on this image. The balloon can be successfully advanced into the Eustachian tube without any issues. The endoscope can be used to safely withdraw the inflating balloon. No device-related damage occurs during the test. The regression process after the test can be observed in the Eustachian tube and nasal cavity.

#### Procedure is effective

The advancement of the supporting balloon is moderately laborious, with moderate handling required. The tissue condition in the human body where the lens reaches can be partially observed during the endoscope push process, or can be observed after processing, allowing for adjustment of the push direction and completion of the push based on this image. The endoscope can be used safely to withdraw the inflating balloon. No device-related damage occurs during the test. The regression process after the test can be observed in the Eustachian tube and nasal cavity.

#### Procedure is ineffective

The advancement process of the supporting balloon is laborious, possibly because of a sharp front end of the combined instrument, or an inability to observe the tissue condition in the human body where the lens reaches during the endoscope push process. The Eustachian tube video endoscope cannot be adjusted and advanced properly. The supporting balloon cannot be withdrawn using the endoscope, or the lens body is broken (non-human factors), the balloon falls off during the operation, the balloon leaks or breaks, or the cavity of the cadaver head is severely damaged during the operation. The regression process after the test cannot be observed in the Eustachian tube and nasal cavity.

### Statistics

Statistical analysis was conducted with GraphPad Prism version 8.0 statistical software. A two-tailed paired-samples test and chi-square test were performed, with *p* < 0.05 indicating statistical significance.

## Results

### Study cohort clinical features

A total of nine cadavers (seven males and two females, aged 30–70 years) who had previously undergone tympanostomy tube insertion, underwent Eustachian tube video endoscope examination and balloon dilatation Eustachian tuboplasty of the cartilaginous portion of their Eustachian tube, on 10 October 2020. Use of a 45° catheter was required for six of the nine Eustachian tubes; three procedures required both a 45° and a 60° catheter, and one necessitated the use of a 30°, a 45° and a 60° catheter.

### Endoscope and supporting balloon

**Figure 2.**
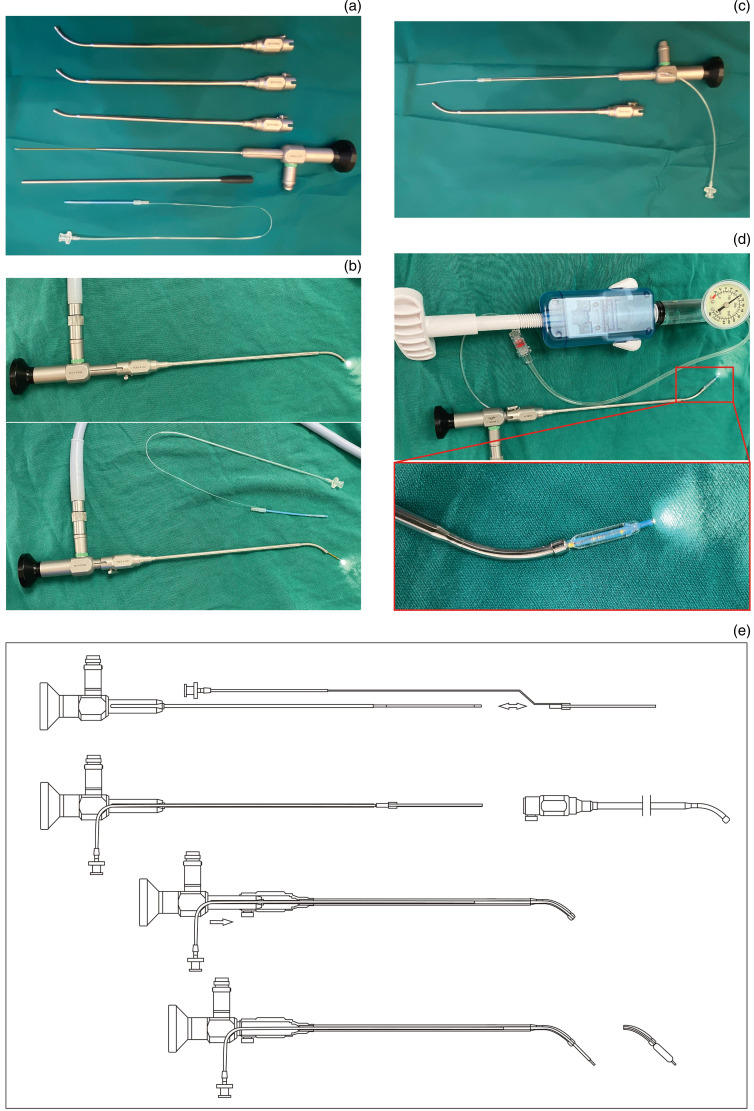
Structure of the Eustachian tube video endoscope and its supporting balloon. (a) The shape characteristics of the Eustachian tube video endoscope. (b) The catheter changing the angle of the Eustachian tube video endoscope soft segment. (c) Combination mode of Eustachian tube video endoscope and its supporting balloon. (d) The angle of the supporting balloon can change with the Eustachian tube video endoscope according to the catheter used. (e) Diagram of the equipment.

The Eustachian tube video endoscope consists of a hard segment and a soft segment (Figure 2a). The catheter has three angles, 30°, 45° and 60°, which can be adjusted to change the angle of the endoscopic soft segment to adapt to different Eustachian tubes (Figure 2b). The supporting balloon can be sleeved on the Eustachian tube video endoscope soft segment (Figure 2c), and used with the catheter to advance the matching balloon along the Eustachian tube video endoscope soft segment to the appropriate position of the Eustachian tube (Figure 2d). The schematic diagram and operational illustration of this equipment are shown in Figure 2e.

### Practical application

**Figure 3.**
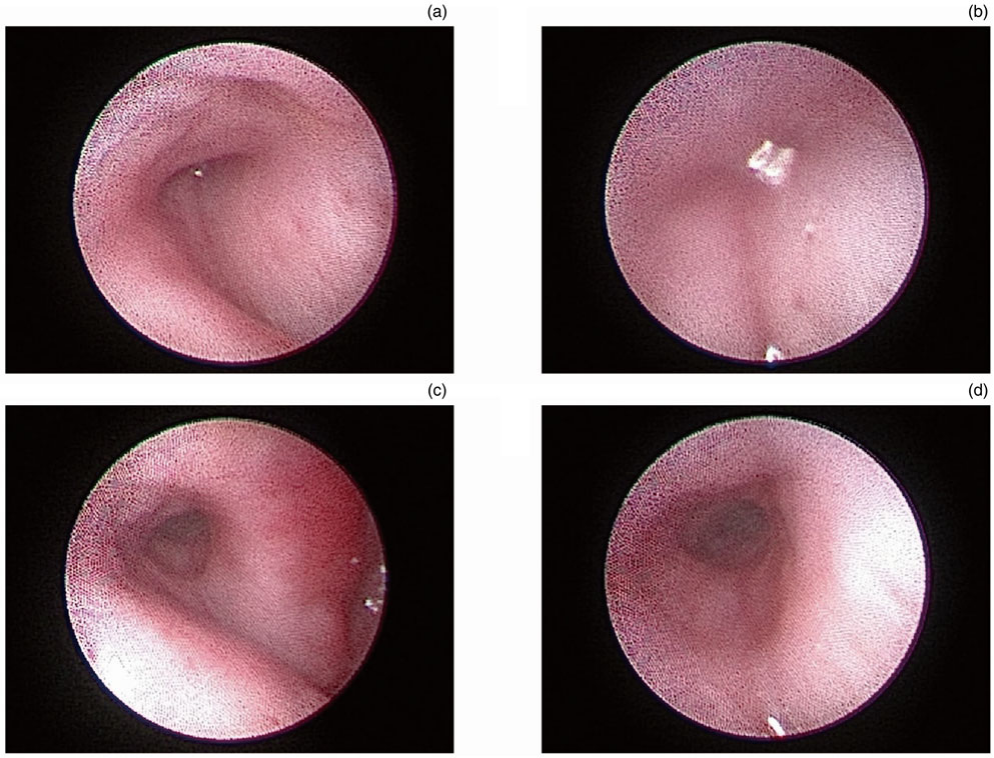
The pre- and post-operative Eustachian tube on Eustachian tube video endoscopy. (a) The pharyngeal ostium of the Eustachian tube on Eustachian tube video endoscopy. (b) The narrowest position of the Eustachian tube on Eustachian tube video endoscopy. (c) The post-operative Eustachian tube cavity on Eustachian tube video endoscopy. (d) The narrowest segment of the post-operative Eustachian tube on Eustachian tube video endoscopy.

The Eustachian tube video endoscope instrument was inserted into the Eustachian tube to observe the position reached, with the supporting balloon installed in the front section (soft section) of the endoscope. We encountered no technical difficulties in identifying the pharyngeal Eustachian tube orifice in the nine human cadavers. The endoscope tip was advanced into the pharyngeal ostium of the Eustachian tube, and the Eustachian tube cavity was examined (Figure 3a). Then, the balloon catheter was gently advanced through the working channel with the endoscope until the narrowest position of the Eustachian tube was reached (Figure 3b).

Once the balloon was correctly positioned, dilatation was implemented using sodium chloride solution up to a pressure of 10 bars for 2 minutes. Then, the solution from the balloon was aspirated, and the catheter, endoscope and balloon were carefully removed under endoscopic control.

In all cases, the catheter and balloon were correctly positioned, as confirmed by the endoscope video. After being correctly positioned in the ostium, the balloon catheter was advanced by an assistant without resistance. After the balloon was dilated, it was removed from the soft segment of the Eustachian tube video endoscope, and then the Eustachian tube video endoscope was pushed forward to observe the state of the Eustachian tube cavity. The narrowest segment of Eustachian tube was clearly observed (Figure 3c–d).

Post-operative results are summarised in Table 1. Two pre-operative Eustachian tubes were open or semi-open (22.22 per cent), and the others were closed (77.78 per cent). All post-operative Eustachian tubes were open. After the operation, the Eustachian tube cavity was opened, and the Eustachian tube video endoscope could enter and exit the Eustachian tube cavity very smoothly for observation. Moreover, the operation did not damage the mucosa of the Eustachian tube cavity. None of the procedures were terminated because of operation-related accidents.

**Table 1.** Post-operative results

Parameter	Pre-operative	Post-operative	*P*-value
ET morphology (*n* (%))			0.0007
– ET open	2 (22.22)	9 (100)	
– ET closed	7 (77.78)	0 (0)	
ET observation (*n* (%))			<0.0001
– Whole ET observed	0 (0)	9 (100)	
– ET observed in part	9 (100)	0 (0)	
Cavity damage? (*n* (%))			>0.9999
– Yes	0 (0)	0 (0)	
– No	9 (100)	9 (100)	
Operative accidents? (*n* (%))			>0.9999
– Yes	0 (0)	0 (0)	
– No	9 (100)	9 (100)	
Time taken for balloon placement (mean ± SD; minutes)		2.22 ± 1.20	
Procedure effectiveness evaluation (*n* (%))			
– Significantly effective		8 (88.89)	
– Effective		1 (11.11)	
– Invalid		0 (0)	

ET = Eustachian tube; SD = standard deviation

With the application of Eustachian tube video endoscope, it took 2.22 ± 1.20 minutes (mean ± standard deviation) to position the balloon, and the whole operation process was easy to implement. Balloon dilatation Eustachian tuboplasty was shown to be significantly effective for eight Eustachian tubes (88.89 per cent) and effective for one Eustachian tube (11.11 per cent).

## Discussion

Clinical studies have showed that balloon dilatation Eustachian tuboplasty is an effective and efficient treatment for diseases caused by Eustachian tube dysfunction.^13,14,19–21^ However, some studies have reported that balloon dilatation Eustachian tuboplasty may not always yield ideal results. For instance, balloon placement may result in Eustachian tube injury^22–24^ associated with failure to accurately observe the position of the balloon during advancement, and failure to promptly stop the procedure when mucosal damage occurs. Although computed tomography (CT) has been used in some studies to determine the position of the balloon,^23,25^ it is not possible to use CT to guide balloon placement during surgery.^26^ Some studies have also used navigation for balloon dilatation Eustachian tuboplasty, but navigation is not available in all hospitals and may not be easily applicable on a wide scale.^24,27^ The present study aimed to verify a new design of Eustachian tube video endoscope that can be used to directly inspect the Eustachian tube, observe the correct placement of the balloon, and examine the Eustachian tube cavity after balloon dilatation Eustachian tuboplasty.

Patients with Eustachian tube dysfunction often exhibit submucosal inflammatory infiltration and even follicle formation in the Eustachian tube prior to surgery. Lymphatic follicles have been described as tonsils of the fallopian tube or Gerlach's tonsil, and are part of the Waldeyer's ring of lymphoid tissue surrounding the oropharynx and nasopharynx. Some studies have shown that adenoid hypertrophic tissue with ‘cobblestoning’ typically corresponds to lymphoid hyperplasia.^28,29^ Many patients who require balloon dilatation Eustachian tuboplasty may also suffer from conditions such as allergic rhinitis, chronic rhinosinusitis, local biofilms and oedema of pharyngeal orifice,^19,28,30,31^ which can make balloon placement challenging. Therefore, it is necessary to develop an instrument that can directly inspect and visualise the Eustachian tube cavity for accurate balloon placement.

In traditional balloon dilatation Eustachian tuboplasty, the suitable position of the balloon is not directly observed during advancement, which can make it challenging for the balloon to enter the Eustachian tube in many cases.^23^ Moreover, traditional balloon dilatation Eustachian tuboplasty may be associated with complications such as mucosal injury, pathological Eustachian tube dysfunction, epistaxis (nosebleeds), carotid artery injury (tear or pseudoaneurysm), subcutaneous emphysema from mucosal injury, infection, and anaesthesia-related complications.^22^ These complications often arise from failure to accurately observe the position reached by the balloon.

Prior to the operation, only a part of the Eustachian tube cavity at the pharyngeal orifice is observable in most cases, even when both Eustachian tubes are partially open. This means that observation of the Eustachian tube is limited without the use of the Eustachian tube video endoscope. Although the structure of the supporting balloon used in Eustachian tube video endoscopy is different from that of a traditional balloon, their working principle is the same. In this study, all Eustachian tubes were open after balloon dilatation Eustachian tuboplasty using the supporting balloon, demonstrating that the expansion ability of the supporting balloon for Eustachian tubes is comparable to that of the traditional balloon. With the guidance of the Eustachian tube video endoscope, the average time taken for the balloon to reach the suitable position was only 2.22 minutes, except for the first operation which took 5 minutes, and the others did not exceed 3 minutes. The success rate of complete Eustachian tube opening after balloon dilatation Eustachian tuboplasty was 100 per cent in the nine cases. Moreover, the entire process was completed without any operative accident related termination, and no mucosal damage was observed before or after balloon dilatation Eustachian tuboplasty, indicating the high safety of the Eustachian tube video endoscope procedure. After balloon dilatation Eustachian tuboplasty, the entire Eustachian tube cavity could be clearly observed with the Eustachian tube video endoscope, enabling effective evaluation of the Eustachian tube after the procedure.

In terms of the angle of the external catheter used in the operation, a 45° catheter was used for six out of the nine Eustachian tubes (66.67 per cent), 45° and 60° catheters were used for two of the nine Eustachian tubes (22.22 per cent), and 30°, 45° and 60° catheters were used for only one Eustachian tube (11.11 per cent); this is consistent with data indicating that the Eustachian tube axis with reference to the sagittal plane is approximately 42°.^32,33^ The design of multiple catheters in the new Eustachian tube video endoscope can cater to the needs of different patients during the procedure.

If the Eustachian tube video endoscope and its supporting balloon are demonstrated to be clinically effective, with sustained benefits over time, they could serve as a useful and minimally invasive alternative for patients with Eustachian tube dysfunction. The findings of the present study, along with previous studies, suggest that the Eustachian tube video endoscope may result in lower injury compared to a traditional balloon, particularly in patients with Eustachian tube dysfunction and those with nasopharyngeal carcinoma after radiotherapy, who may not be suitable candidates for traditional balloon treatment.

## Conclusion

Use of the Eustachian tube video endoscope technique, with its supporting balloon, offers a promising and minimally invasive approach for treating Eustachian tube dysfunction. The Eustachian tube video endoscope procedure allows for direct observation of the balloon as it is advanced, reducing the risk of complications associated with traditional balloon techniques. The expansion ability of the supporting balloon for Eustachian tubes is comparable to that of traditional balloons, as demonstrated by the successful opening of all Eustachian tubes in the study. The use of the Eustachian tube video endoscope also allows for better visualisation of the entire Eustachian tube cavity before and after the procedure, facilitating more effective evaluation. Additionally, the multiple catheter design of the Eustachian tube video endoscope allows for flexibility in choosing the appropriate angle for catheter insertion.

The Eustachian tube video endoscope technique may be particularly beneficial for patients with Eustachian tube dysfunction and those with nasopharyngeal carcinoma after radiotherapy, who may not be suitable candidates for traditional balloon treatments because of increased risks of complications. However, further clinical studies are needed to validate the clinical effectiveness and long-term benefits of the Eustachian tube video endoscope. Overall, the Eustachian tube video endoscope procedure shows promise as a safe and effective alternative for treating Eustachian tube dysfunction, with potential advantages over traditional balloon techniques; the technique warrants further investigation and consideration in clinical practice.

- The reported outcomes and complications of Eustachian tube balloon surgery are wide-ranging
- This study evaluated the feasibility and safety of a Eustachian tube video endoscope with supporting balloon for the treatment and examination of Eustachian tube dysfunction
- The Eustachian tube video endoscope and supporting balloon technique was used on nine corpses
- The technique was easy to perform, with no damage to essential structures including the Eustachian tube cavity observed
- Eustachian tube cavity examination using the Eustachian tube video endoscope and supporting balloon is a feasible, safe procedure
